# Right mainstem intubation improves transoesophageal echocardiographic guidance during mitral transcatheter edge-to-edge repair

**DOI:** 10.1093/ehjcr/ytag767

**Published:** 2026-10-19

**Authors:** Yasuhisa Nakao, Kazuhisa Nishimura, Katsuji Inoue, Osamu Yamaguchi

**Affiliations:** Department of Cardiology, Pulmonology, Hypertension and Nephrology, Ehime University Graduate School of Medicine, Shitsukawa 454, Toon 791-0295, Ehime, Japan; Department of Cardiology, Pulmonology, Hypertension and Nephrology, Ehime University Graduate School of Medicine, Shitsukawa 454, Toon 791-0295, Ehime, Japan; Department of Cardiology, Pulmonology, Hypertension and Nephrology, Ehime University Graduate School of Medicine, Shitsukawa 454, Toon 791-0295, Ehime, Japan; Department of Cardiology, Pulmonology, Hypertension and Nephrology, Ehime University Graduate School of Medicine, Shitsukawa 454, Toon 791-0295, Ehime, Japan

**Keywords:** Mitral transcatheter edge-to-edge repair, Transoesophageal echocardiography, Right mainstem intubation, Pulmonary interposition, Mitral regurgitation

## Summary

Right mainstem intubation improved transoesophageal echocardiographic visualization limited by pulmonary interposition during mitral transcatheter edge-to-edge repair, enabling successful device implantation in a patient with severe degenerative mitral regurgitation.

## Case description

A man in his 70s was admitted for evaluation of cholangiocarcinoma and developed acute heart failure. Transthoracic echocardiography showed P2 prolapse with chordal rupture, resulting in severe mitral regurgitation (MR). Because of the elevated surgical risk and active malignancy, mitral transcatheter edge-to-edge repair (M-TEER) targeting A2–P2 was planned.

Intra-procedural transoesophageal echocardiography (TEE) showed inadequate visualization of the true intercommissural view, particularly the medial mitral valve. Despite repeated attempts using standard and deeper probe positions (*Figure 1A*; Supplementary material online*, Video S1*), mitral valve morphology, device orientation, and leaflet grasping site could not be reliably assessed, and discontinuation was considered.

**Figure 1:**
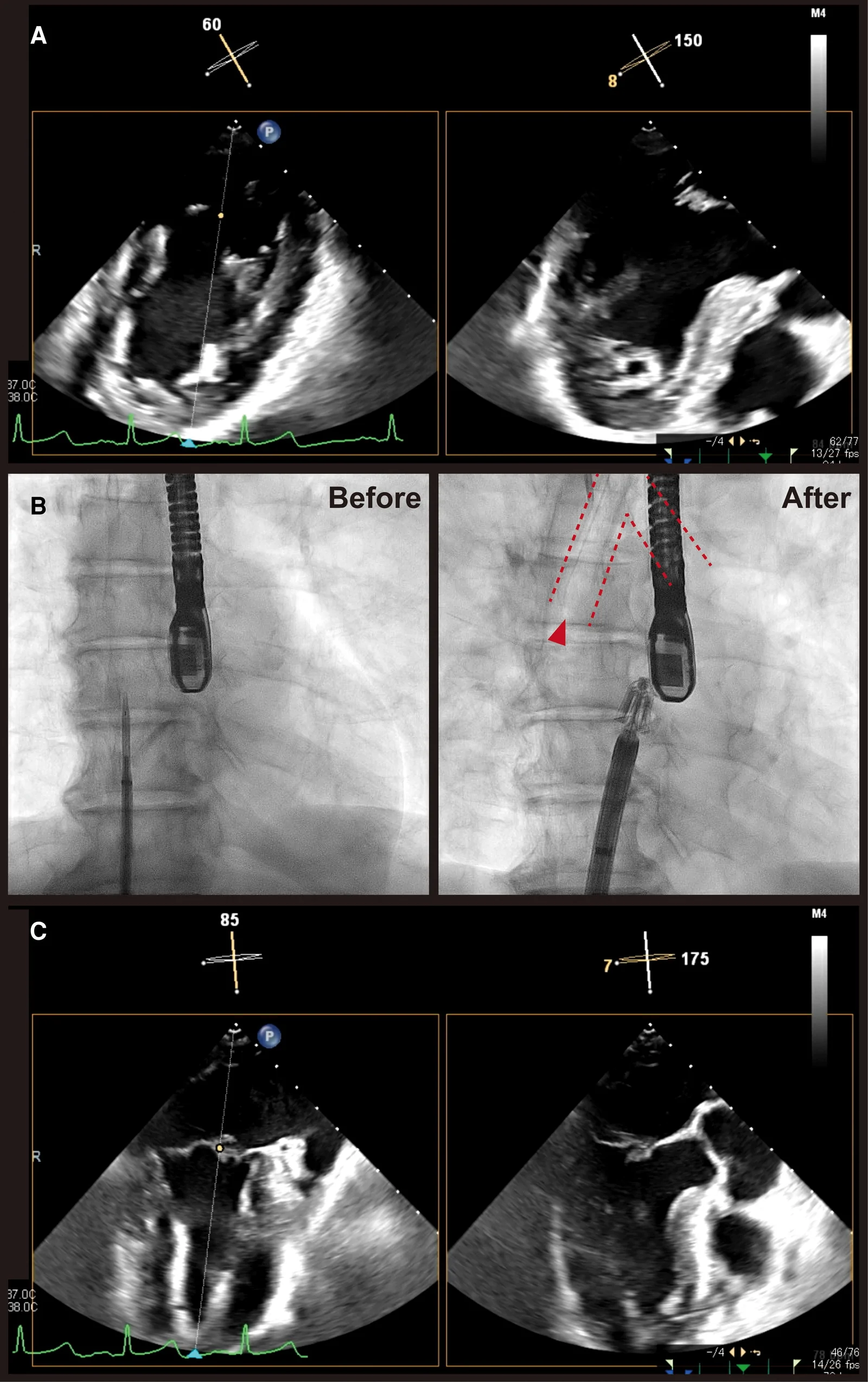
Right mainstem intubation during mitral transcatheter edge-to-edge repair. (*A*) Transoesophageal echocardiography before right mainstem intubation, showing limited visualization of the medial mitral valve. (*B*) Fluoroscopy before and after right mainstem intubation. Dashed lines indicate the bronchi, and the arrowhead indicates the tube tip. (*C*) Transoesophageal echocardiography after right mainstem intubation, showing improved visualization of the medial mitral valve and device orientation for implantation.

Pulmonary interposition was suspected to be the main cause of acoustic interference. After discussion with anaesthesiologists, the endotracheal tube was advanced into the right main bronchus under fluoroscopic guidance as a simple intra-procedural airway manoeuvre (*Figure 1B*). Oxygenation remained stable. After right mainstem intubation, TEE image quality improved, especially for the medial aspect of the mitral valve (*Figure 1C*; Supplementary material online, *Video S2*). This enabled reassessment of valve morphology, device orientation, and leaflet grasping, and one PASCAL Ace device was implanted at A2–P2. Residual MR prompted placement of a second device, increasing the mean transmitral gradient from 3.1 to 9.4 mmHg. The second device was retrieved, and the procedure was completed with a single device. Mitral regurgitation was reduced from severe to mild, with a final mean transmitral gradient of 3.5 mmHg (see Supplementary material online, *Videos S3* and *S4*). The patient has remained free from worsening of heart failure during more than 6 months of outpatient follow-up.

Previous reports used one-lung ventilation with bronchial blockers to improve transseptal imaging,^1^ predominantly transthoracic guidance,^2^ or posterior leaflet TEE visualization.^3^ This case suggests that fluoroscopically guided right mainstem tube advancement without a bronchial blocker may be a practical intra-procedural rescue option to restore medial intercommissural TEE visualization.

## Lead author biography


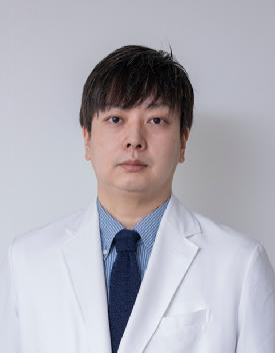
Yasuhisa Nakao, MD, PhD, is a cardiologist and physician-scientist at Ehime University Graduate School of Medicine, Japan. His clinical interests include heart failure, structural heart disease, cardiovascular imaging, and transcatheter interventions for valvular heart disease. His research interests encompass heart failure, cardiovascular genetics, vascular biology, and vascular calcification, with a particular interest in the mechanisms underlying cardiovascular disease. He is actively involved in clinical practice, translational research, and medical education.

## Supplementary Material

ytag767_Supplementary_Data

## Data Availability

The data supporting this case report are included in the article and its Supplementary material. Additional patient-level data are not publicly available to protect patient confidentiality.
