# Supplementary material for: Right mainstem intubation improves transoesophageal echocardiographic guidance during mitral transcatheter edge-to-edge repair
Source: Eur Heart J Case Rep. 2026 Oct 19;10(11):ytag767. doi: 10.1093/ehjcr/ytag767 (PMC13738064; doi:10.1093/ehjcr/ytag767)
Supplement: Supplementary file 1 [file ytag767_supplementary_data.zip › M-TEER_Supplementary video legends.docx]

**Supplementary video legends**

**Supplementary Video S1.** TEE before right mainstem intubation, showing limited visualization of the medial mitral valve.

**Supplementary Video S2.** TEE after right mainstem intubation, showing improved visualization of the medial mitral valve.

**Supplementary Video S3.** Pre-procedural transesophageal echocardiography showing severe mitral regurgitation.

**Supplementary Video S4.** Post-procedural transesophageal echocardiography showing mild residual mitral regurgitation after mitral transcatheter edge-to-edge repair with a single PASCAL Ace device.
